# Whole-Genome Sequencing Analysis of Serially Isolated Multi-Drug and Extensively Drug Resistant *Mycobacterium tuberculosis* from Thai Patients

**DOI:** 10.1371/journal.pone.0160992

**Published:** 2016-08-12

**Authors:** Kiatichai Faksri, Jun Hao Tan, Areeya Disratthakit, Eryu Xia, Therdsak Prammananan, Prapat Suriyaphol, Chiea Chuen Khor, Yik-Ying Teo, Rick Twee-Hee Ong, Angkana Chaiprasert

**Affiliations:** 1 Department of Microbiology Faculty of Medicine, Khon Kaen University, Khon Kaen, Thailand; 2 Research and Diagnostic Center for Emerging Infectious Diseases (RCEID), Khon Kaen University, Khon Kaen, Thailand; 3 Saw Swee Hock School of Public Health, National University of Singapore, Singapore; 4 Department of Microbiology, Faculty of Medicine Siriraj Hospital, Mahidol University, Bangkok, Thailand; 5 NUS Graduate School for Integrative Sciences and Engineering, National University of Singapore, Singapore; 6 National Center for Genetic Engineering and Biotechnology, National Science and Technology Development Agency, Ministry of Science and Technology, Pathum Thani, Thailand; 7 Bioinformatics and Data Management for Research Unit, Office for Research and Development, Faculty of Medicine Siriraj Hospital, Mahidol University, Bangkok, Thailand; 8 Genome Institute of Singapore, Singapore; 9 Department of Statistics and Applied Probability, National University of Singapore, Singapore; 10 Life Sciences Institute, National University of Singapore, Singapore; University of Minnesota, UNITED STATES

## Abstract

Multi-drug and extensively drug-resistant tuberculosis (MDR and XDR-TB) are problems that threaten public health worldwide. Only some genetic markers associated with drug-resistant TB are known. Whole-genome sequencing (WGS) is a promising tool for distinguishing between re-infection and persistent infection in isolates taken at different times from a single patient, but has not yet been applied in MDR and XDR-TB. We aim to detect genetic markers associated with drug resistance and distinguish between reinfection and persistent infection from MDR and XDR-TB patients based on WGS analysis. Samples of *Mycobacterium tuberculosis* (n = 7), serially isolated from 2 MDR cases and 1 XDR-TB case, were retrieved from Siriraj Hospital, Bangkok. The WGS analysis used an Illumina Miseq sequencer. In cases of persistent infection, MDR-TB isolates differed at an average of 2 SNPs across the span of 2–9 months whereas in the case of reinfection, isolates differed at 61 SNPs across 2 years. Known genetic markers associated with resistance were detected from strains susceptible to streptomycin (2/7 isolates), p-aminosalicylic acid (3/7 isolates) and fluoroquinolone drugs. Among fluoroquinolone drugs, ofloxacin had the highest phenotype-genotype concordance (6/7 isolates), whereas gatifloxcain had the lowest (3/7 isolates). A putative candidate SNP in *Rv2477c* associated with kanamycin and amikacin resistance was suggested for further validation. WGS provided comprehensive results regarding molecular epidemiology, distinguishing between persistent infection and reinfection in M/XDR-TB and potentially can be used for detection of novel mutations associated with drug resistance.

## Introduction

Drug resistant tuberculosis (DR-TB), especially multi-drug and extensively drug resistant TB (MDR and XDR-TB), is a serious public health problem causing higher cost of treatment, lower treatment success rates, higher rate of persistent infection and higher mortality rates in TB patients.

Genetic analysis of DR-TB provides rapid information for early management of TB patients. However, genetic markers and mechanisms producing drug resistance are not completely known, especially in the case of second line anti-TB drugs [1]. Although random mutations may occur, comparisons between drug-resistant and susceptible cohorts in large *Mtb* populations provide statistically convincing identification of genetic markers for DR-TB [2]. Serially isolated *Mtb* samples from a single patient showing gradual increase of drug resistance also provides raw material for the study of genetics associated with drug resistance [3–5]. The genetic backbone of the *Mtb* lineage derived from the same clone diminishes the risk of assuming that random mutations are in fact associated with drug resistance. Additional WGS analysis of serially isolated *Mtb* samples from different settings and drug resistance profiles are required to provide the lacking information.

The transmission chain of TB from person to person can be traced by genotyping *Mtb* isolates, indicating whether the transmitted strain derived from the same source or not. Previously, serially isolated *Mtb* clones were investigated for the stability of the markers used for genotyping during intra-patient evolution [6–8]. Whole-genome sequence (WGS) analysis of serially isolated *Mtb* samples has revealed that genetic changes occurring within a single TB patient during the course of infection are as frequent as found among patients [9] and that 0–2 SNPs occur per transmission event [10]. Serially isolated *Mtb* samples from different settings can be investigated for the optimal numbers of SNPs for TB transmission analysis.

When TB re-occurs in a treated patient, this could be due to persistent infection caused by persistence of the same bacilli despite treatment or reinfection caused by a new strain of bacilli. The treatment course can take up to 24 months in cases of M/XDR TB. Consequently, it can be difficult to distinguish between persistent infection and reinfection. Persistent infection is a sign of treatment failure. High-resolution molecular typing, such as IS*6110* RFLP or MIRU-VNTR typing, is required to distinguish between persistent infection and reinfection. Whole-genome sequencing (WGS) is also promising for this purpose. WGS has been used for direct detection of *Mtb* from sputum samples [11], detection of mixed infections [12] and outbreak investigation [13, 14]. However, WGS has only been used once to distinguish between reinfection and persistent infection [15]. No study has applied WGS analysis to distinguish between reinfection and persistent infection in MDR- and XDR-TB yet. The high selective pressure from anti-TB drugs and higher mutability of the Beijing lineage of *Mtb* [16] influences the mutation rate which can be investigated using WGS analysis of serially isolated samples.

In this study, we analyzed the WGS of *Mtb* serially isolated from MDR and XDR-TB patients from Thailand. The genetic markers associated with drug resistance and comparative genomic analysis between cases of reinfection and persistent infection were investigated.

## Materials and Methods

### *Mtb* isolates and setting

Seven *Mtb* isolates obtained from two MDR patients and one XDR-TB case were retrieved from stock cultures of clinical isolates deposited at the Drug-Resistant Tuberculosis Research Fund, Faculty of Medicine Siriraj Hospital, Mahidol University, Bangkok, Thailand, between 2007 and 2012. The study protocol was approved by the Ethical and Scientific Committees of the Faculty of Medicine Siriraj Hospital, Mahidol University (EC No. Si 029/2557).

### Drug susceptibility test

Phenotypic drug susceptibility tests for anti-TB drugs were performed using standard proportional methods [17] on Middlebrook (M) 7H10 agar plates. Drug concentrations of 0.2 mg/l for isoniazid, 1.0 mg/l for rifampicin, 5.0 mg/l for ethambutol and ethionamide, 6.0 mg/l for amikacin and kanamycin, and 2.0 mg/l for streptomycin, *p*-aminosalicylic acid, ofloxacin, levofloxacin, moxifloxacin and gatifloxacin were used.

### Culture of mycobacteria and DNA extraction

All *Mtb* isolates were sub-cultured onto LJ media and incubated at 37°C for four weeks. Genomic DNA from *Mtb* isolates was extracted from multiple loopfuls of *Mtb* colonies using the cetyl-trimethyl-ammonium bromide-sodium chloride method [18].

### Classical genotyping

Spoligotyping was performed according to the manufacturer’s instructions (Ocimum Biosolutions, India) as previously described [19]. Twenty four loci MIRU-VNTR typing was performed according to the standard method using the multiplex and simplex PCR according to the study published previously [20].

### Whole-genome sequencing

Sequencing of the *Mtb* isolates was performed at the Genome Institute of Singapore (GIS), Singapore. Genomic libraries were prepared according to the recommendations of the TrueSeq DNA sample preparation kit (Illumina, San Diego, CA). The library pools were subjected to paired-end sequencing on a MiSeq platform (Illumina) generating 250-bp read lengths. The sequence data have been deposited in the Sequence Read Archive (SRA) with the study accession No. SRP071184.

### Bioinformatics and data analysis

#### Mapping and variant calling

The overall quality of sequence read was checked using FastQC version 0.11.3 [21]. All sequences with an average quality score above 36 were retained. Reads shorter than 36 bp and possible contaminating adaptor sequences were excluded using Trimmomatic version 0.33 [22]. Paired-end raw reads of each isolate were mapped to the *Mtb* H37Rv reference genome (GenBank accession number: NC_000962.3) using BWA MEM version 0.7.12 [23]. Samtools version 0.1.19 [24] was used for SAM-BAM format conversion and sorting of mapped sequences. Local realignment of the mapped reads was performed using GATK version 3.4.0 [25]. The stat reports were generated using GATK and Samtools indicating that the average coverage of the mapped sequences was 64X (±3.51) and the average mapping rate of the sequences was 99.36% (±0.22%). Variants, including single nucleotide polymorphisms (SNPs), were called using GATK and Samtool tools [24]. Variant sites were filtered based on the following criteria: mapping quality ≥50 (-C in Samtools calling), base quality/base alignment quality ≥20 (-Q in Samtools calling), ≥10 reads or ≤2,000 reads (-d in Samtools filter) covering each site. To maximize specificity, the called variants were selected from the intersection of those identified by Samtools and GATK. The snpEff version 4.1 [26] was used for variant annotation. Variants identified in the repetitive regions and paralogous gene families (PE, PPE, PE-PGRS, integrase, transposase and phage-related genes) were discarded. Additionally, heterozygous SNPs with allelic frequencies of <75% or read-depth <10 reads were excluded. Those remaining and satisfying all the above criteria were regarded as high-confidence SNPs.

#### Detection of genetic markers associated to drug resistance

Genetic markers associated with drug-resistant TB were detected using TB-Profiler [27], PhyResSE [28], KvarQ [29] and PhyTB [30]. Novel resistance-associated marker candidates were determined by comparative genomics between serial isolates of *Mtb* which had exhibited a change from susceptible to resistant phenotype. Selected SNPs identified were further validated by PCR/Sanger sequencing. Descriptive statistics were used to analyze the data.

#### Protein structure analysis

The 267 amino-acid sequence (including a 133 amino-acid flanking sequence) of *Rv2477c*, retrieved from an online database, was analyzed for the effect of SNPs on protein structure. PhyRe2 [31] was used to predict the protein structure. TM-align [32] and Chimera version 1.10.2 (https://www.cgl.ucsf.edu/chimera/) were used to compare the protein structure between wild-type and mutant.

#### *In silico* spoligotyping, LSPs and SNP-barcode typing analysis

The *in silico* analysis of spoligotyping was performed using SpolPred [33] and SpoTyping [34]. The lineages of *Mtb* based on SNPs-barcode typing [35] were classified using TB-Profiler [27]. The presence or absence of specific regions of difference was analyzed by mapping reads to the RD sequences using BWA MEM version 0.7.12 [23] and the alignments analyzed manually using Samtools read depth.

#### Phylogenetic analysis

Phylogenetic analysis of the 1,890 high-confidence SNPs identified among seven *Mtb* isolates was performed based on the maximum likelihood (ML) method using MEGA-6 [36] with a general time-reversible (GTR) model of nucleotide substitution and a gamma model of rate heterogeneity. The phylogenetic tree was constructed based on 1,000 bootstrap replicates.

#### Data analysis

Descriptive statistics were used to describe the characteristics of the *Mtb* isolates, including drug susceptibility profiles and genetic characteristics. Fisher's exact test was used to compare between the numbers of *Mtb* isolates with phenotypic-genotypic discordance between pairs of anti-TB drugs. SPSS version 16 (SPSS Inc., Illinois, USA) was used. For all analyses, a p value <0.05 was considered to be statistically significant.

## Results

### Characteristics of drug resistant *Mtb* isolates from DR-TB patients

Drug resistant *Mtb* isolates (n = 7) were isolated from three patients. Patient I was diagnosed with XDR-TB and 2 isolates were obtained 70 days apart during the course of treatment. Patient II was a case of preXDR-TB and 2 isolates were obtained 9 months apart during the course of treatment. Patient III was initially identified as MDR-TB. One isolate was obtained at this stage. The patient subsequently developed pre-XDR-TB during continuing treatment and 2 further isolates were obtained, 25 months and 33 months after the initial isolate (Table 1). Demographic data from these patients are not available.

**Table 1:** Drug susceptibility profile based on phenotype and whole-genome sequencing analysis of serially isolated *M*. *tuberculosis*.

Subjects	Codes	Date of isolation	DR	DST	Drug resistant profiles																		
					INH	RIF	EMB	PZA	SM	FQ.gr	OFX	MOX	GAT	LEV	AG.gr	AK	K	Cm	ETO	PAS	Lzd	Cfz	Bdq
Patient I																							
Isolate I	WBB259 (DS16220)	9/11/2007	XDR	Phenotype	R	R	R	NA	S	NA	R	R	R	R	NA	R	R	NA	S	S	NA	NA	NA
				WGS	R ^1,2,3^	R ^1,2,3,4^	R ^1,2,3^	R ^1,3,4^	R ^1,4^	R ^1,2,3^	NA	NA	NA	NA	S	R ^1,2,3^	R ^1,2,3^	R ^1,2^	S	R^1^	S	S	S
Isolate II	WBB260 (DS16780)	19/1/2008	XDR	Phenotype	R	R	R	NA	S	NA	R	R	R	R	NA	R	R	NA	S	S	NA	NA	NA
				WGS	R ^1,2,3^	R ^1,2,3,4^	R ^1,2,3^	R ^1,3,4^	R ^1,4^	R ^1,2,3^	NA	NA	NA	NA	S	R ^1,2,3^	R ^1,2,3^	R ^1,2^	S	R^1^	S	S	S
Patient II												#				+	+						
Isolate I	WBB270 (DS19048)	31/10/2008	PreXDR	Phenotype	R	R	R	NA	R	NA	R	R	S	R	NA	S	S	NA	S	R	NA	NA	NA
				WGS	R ^1,2,3^	R ^1,2,3,4^	R ^1,2,3^	S	R ^1,2,3,4^	R ^1,2,3^	NA	NA	NA	NA	S	S	S	S	S	S	S	S	S
Isolate II	WBB273 (DS21277)	24/7/2009	PreXDR	Phenotype	R	R	R	NA	R	NA	R	S	S	R	NA	R	R	NA	S	R	NA	NA	NA
				WGS	R ^1,2,3^	R ^1,2,3,4^	R ^1,2,3^	S	R ^1,2,3,4^	R ^1,2,3^	NA	NA	NA	NA	S	S	S	S	S	R^1^	S	S	S
Patient III												+	+	+									
Isolate I	WBB274 (DS21644)	12/9/2009	MDR	Phenotype	R	R	R	NA	R	NA	S	S	S	S	NA	S	S	NA	R	S	NA	NA	NA
				WGS	R ^1,2,3,4^	R ^1,2,3,4^	R ^1,2,3^	R ^3^	R ^1,3^	R ^1,2,3^	NA	NA	NA	NA	S	S	S	S	R ^1,2^	R^1^	S	S	S
Isolate II	WBB280 (DS29147)	14/10/2011	PreXDR	Phenotype	R	R	R	NA	R	NA	R	S	S	S	NA	S	S	NA	S	S	NA	NA	NA
				WGS	R ^1,2,3^	R ^1,2,3,4^	R ^1,2,3,4^	R ^1,2,4^	R ^1,2,3,4^	R ^1,2,3^	NA	NA	NA	NA	S	S	S	S	S	S	S	S	S
Isolate III	WBB284 (DS31231)	15/6/2012	PreXDR	Phenotype	R	R	R	NA	R	NA	R	R	R	R	NA	S	S	NA	S	S	NA	NA	NA
				WGS	R ^1,2,3^	R ^1,2,3,4^	R ^1,2,3,4^	R ^1,2,4^	R ^1,2,3,4^	R ^1,2,3^	NA	NA	NA	NA	S	S	S	S	S	S	S	S	S

Drug susceptibility test (DST) of WGS: ^1^TB-Profiler, ^2^PhyResSE, ^3^KVarQ, ^4^PhyTB. VCF file from Samtools calling were used for PhyTB. The result of unspecified aminoglycoside (AG gr.) was from PhyTB only. NA: not available (the gene mutation of particular drug is not included), #: Reversion from resistant to susceptible phenotype, +: Conversion from susceptible to resistant phenotype, DR: drug resisatnce, INH: isoniazid, RIF: rifampicin, EMB: ethambutol, PZA: pyrazinamide, SM: streptomycin, FQ gr.: unspecified fluoroquinolones, OFX: ofloxacin, MOX: moxifloxacin, GAT: gatifloxacin, LEV: levofloxacin, AK: amikacin, K: kanamycin. Cm: capreomycin, ETO: ethionamide, PAS: *p*-aminosalicylic acid, Lzd: linezolid, Cfz: clofazimine, Bdq: bedaquiline. The genetic markers from bioinformatics tools are available for the whole fluoroquinolones but not for the specific drugs, i.e. OFX, MOX, GAT and LEV).

Drug susceptibility patterns of the *Mtb* isolates are shown in Table 1. Of the 17 drugs and 2 unspecified drug groups tested, phenotypic DST results were available for 12. A stable drug-resistant phenotype was found in the 2 isolates of Patient I. The development of susceptible-to-resistant phenotypes for kanamycin and amikacin was found in Patient II and for fluoroquinolones (moxifloxacin, gatifloxacin and leveofloxacin) in Patient III. A resistant-to-susceptible change for moxifloxacin was seen in Patient II. The DNA sequence of *gyrase A* (*gyrA)*–the gene associated with fluoroquinolone resistance—was obtained using Sanger sequencing and showed perfect concordance to the WGS result (data not shown).

### Genotyping analysis of drug resistant *Mtb* isolates

The spoligotyping patterns of *Mtb* isolates from Patient I indicate a Manu-ancestor, whereas the isolates from Patient II and Patient III belong to the Beijing lineage. The 24-loci MIRU-VNTR analysis demonstrated identical genotypes of serial isolates within each patient in Patients I and II. In Patient III, loci VNTR4052 and VNTR4120 in the initial isolate (WBB274 (DS21644)) differed from the two later isolates from this patient, suggesting reinfection (Table 2).

**Table 2:** Genotypes of drug resistant *M*. *tuberculosis* based on spoligotpying and 24 loci MIRU-VNTR.

Patients	Spoligotyping	SNP barcode (62 SNPs)*	24 loci MIRU-VNTR																							
			424	577	580	802	960	1644	1955	2163b	2165	2401	2996	3192	3690	4052	4156	1982	2074	2163a	2372	3155	3232	3336	3820	4120
Patient I																										
WBB259 (DS16220)	777777777777771	Lineage 2.1	4	4	2	3	8	2	4	6	5	2	7	4	1	1	1	5	2	18	3	4	11	10	11	2
WBB260 (DS16780)	777777777777771	Lineage 2.1	4	4	2	3	8	2	4	6	5	2	7	4	1	1	1	5	2	18	3	4	11	10	11	2
Patient II																										
WBB270 (DS19048)	000000000003771	Lineage 2.2.1	4	4	2	3	3	3	5	4	4	4	7	5	3	8	2	8	3	18	3	4	14	7	9	10
WBB273 (DS21277)	000000000003771	Lineage 2.2.1	4	4	2	3	3	3	5	4	4	4	7	5	3	8	2	8	3	18	3	4	14	7	9	10
Patient III																										
WBB274 (DS21644)	000000000003771	Lineage 2.2.1	4	4	0	4	3	3	5	5	4	4	7	5	4	**5**	2	6	3	5	3	4	14	7	14	**10**
WBB280 (DS29147)	000000000003771	Lineage 2.2.1	4	4	0	3	3	3	5	5	4	4	7	5	4	**8**	2	6	3	5	3	4	14	7	14	**11**
WBB284 (DS31231)	000000000003771	Lineage 2.2.1	4	4	0	3	3	3	5	5	4	4	7	5	4	**8**	2	6	3	5	3	4	14	7	14	**11**

The results from MIRU-VNTR (classical technique), spoligotyping (classical and *in silico* technique). Lineage 2.1: East Asian (non Beijing), Lineage 2.2.1: East Asian (Beijing), Spoligotyping was performed using SpolPred and SpoTyping.*; refers to 62 SNPs barcode set for lineage typing of the *M*. *tuberculosis* complex [35]. Bold numbers indicate number of repeats when these differed between serial isolates.

**Fig 1:**
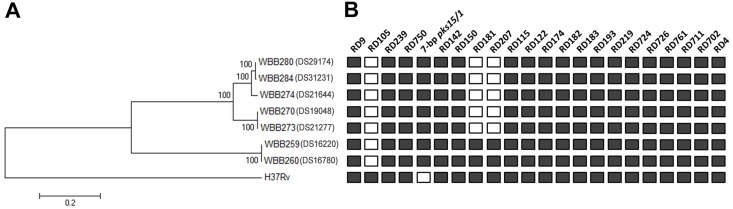
Phylogenetic analysis of serially isolated *M*. *tuberculosis* isolates. **(A)** Phylogenetic tree based on maximum likelihood method using 1,890 high-confidence SNPs and compared to the H37Rv reference genome. The scale bar length represents 0.2 nucleotide substitutions per site. **(B)** The large sequence polymorphism analysis showing the presence/absence of RDs in each isolate.

The genotype of *Mtb* isolates based on spoligotyping, LSPs and robust barcode SNPs showed that those from the same patient were in the same cluster. The barcode SNP-typing and LSP analysis showed that WBB259 (DS16220) and WBB260 (DS16780) belonged to lineage 2.1, exhibiting the RD105 deletion whereas the remaining isolates belonged to lineage 2.2.1 with the RD105, RD181 and RD207 deletions (Table 2 and Fig 1B)

### WGS analysis to distinguish between reinfection and persistent infection of MDR and XDR-TB patients

The phylogenetic analysis based on 1,890 high-confidence SNPs also showed isolates from the same patients to be identical in Patients I and II. In Patient III, the first isolate, WBB274 (DS21644) was distinct from the 2 later isolates obtained from the same patient, suggesting reinfection (Fig 1A).

**Fig 2:**
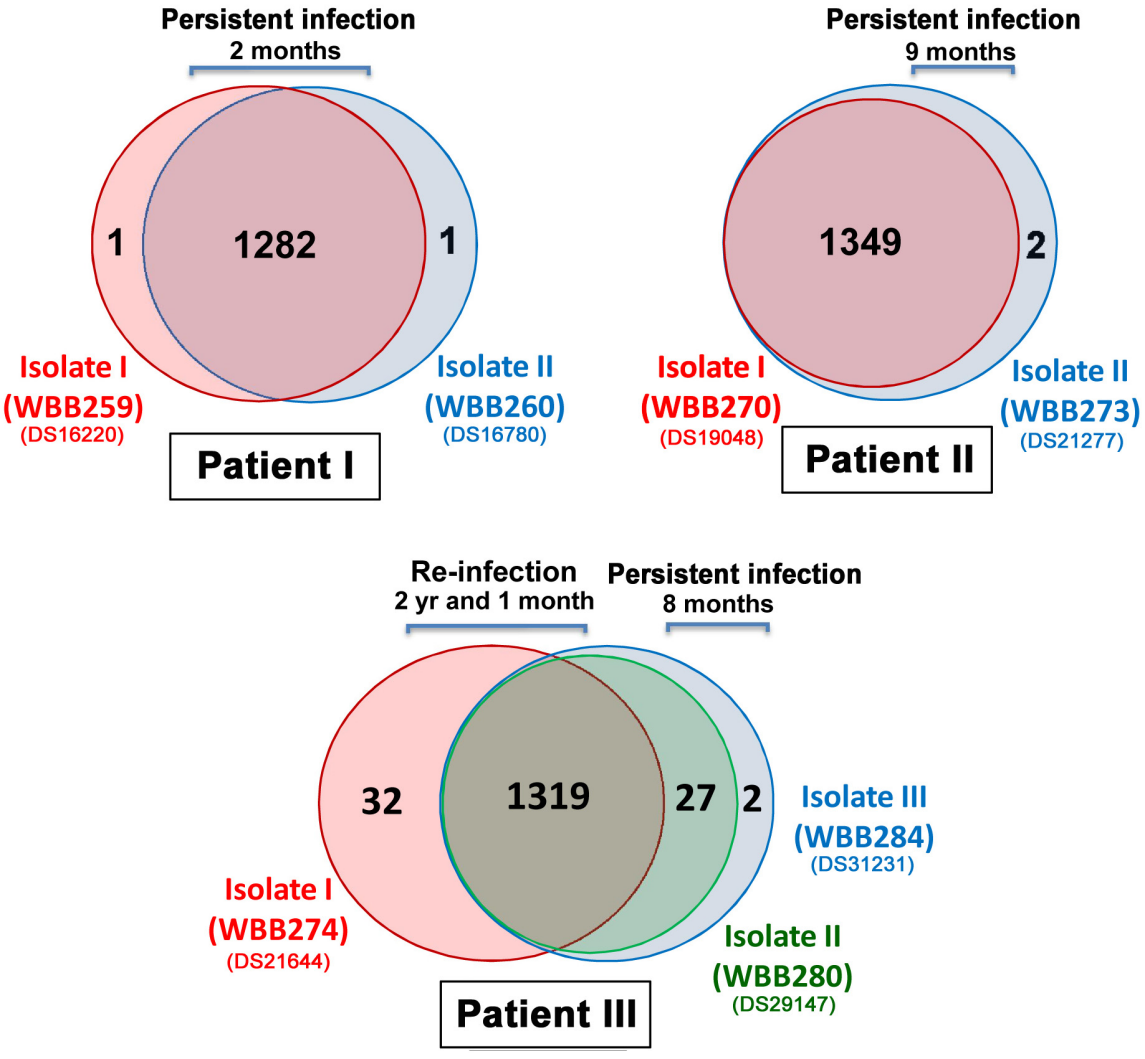
Venn diagram comparing the number of SNPs shared by serially isolated *M*. *tuberculosis* isolates.

On an average, only 2 high-confidence SNPs changed in persistent infection isolates across 2–9 months. This is markedly different to the 61 SNP differences found between the initial and the two later isolates in Patient III (Fig 2).

### Detection of genetic markers associated with drug resistant TB using *in silico* analysis tools

Genetic markers associated with resistance to streptomycin (2/7 isolates), *p*-aminosalicylic acid (3/7 isolates) and fluoroquinolones were detected (using bioinformatics tools) in isolates with phenotypes susceptible to these drugs (Table 3). Among fluoroquinolones, ofloxacin had the highest phenotype-genotype concordance (6/7 isolates) whereas gatifloxcain had the lowest (3/7 isolates), but these differences were not statistically significant (p = 0.266). There is no statistically significant difference among the numbers of *Mtb* isolates with phenotype-genotype discordance from streptomycin, *p*-aminosalicylic acid and fluoroquinolones drugs (p>0.05). In strain WBB273 (DS21277), a kanamycin and amikacin-resistant phenotype developed, but no known genetic marker for resistance to these drugs was detected (Table 3). For some drugs (capreomycin, linezolid, clofazimineand and bedaquiline) for which phenotypic results were not available, WGS analysis found only capreomycin resistance in WBB259 (DS16220) and WBB260 (DS16780) from Patient I.

**Table 3:** SNPs of genes associated with drug-resistant TB.

Drugs	Mutations associated with drug resistance		Drug resistant *M*. *tuberculosis* isolates						
	Genes	Locus	Patient I		Patient II		Patient III		
			WBB259 (DS16220)	WBB260 (DS16780)	WBB270 (DS19048)	WBB273 (DS21277)	WBB274 (DS21644)	WBB280 (DS29147)	WBB284 (DS31231)
INH	Phenotype		R	R	R	R	R	R	R
	*inhA* (Ser94Ala)	1,674,481	X	X	X	X	/	X	X
	*katG* (Ser315Thr)	2,155,168	/	X	X	X	/	X	X
	*katG* (Ser315Asn)	2,155,168	X	/	/	/	X	/	/
RIF	Phenotype		R	R	R	R	R	R	R
	*rpoB*(Ser441Leu)	761,128	X	X	X	X	/	X	X
	*rpoB* (His445Tyr)	761,139	/	/	/	/	X	/	/
	*rpoB* Ser450Leu	761,155	/	/	X	X	X	X	X
	*rpoB* (Glu460Gly)	761,185	X	X	X	X	/	X	X
EMB	Phenotype		R	R	R	R	R	R	R
	*embB* (Met306Val)	4,247,429	X	X	/	/	X	X	X
	*embB* (Met306Ile)	4,247,431	/	/	X	X	/	X	X
	*embB* (Gln497Arg)	4,248,003	X	X	X	X	X	/	/
PZA	Phenotype		NA	NA	NA	NA	NA	NA	NA
	*pncA*(Ile90Ser)	2,288,973	/	/	X	X	X	X	X
	*pncA* (Thr160Pro)	2,288,764	X	X	X	X	/	/	/
SM	Phenotype		S*	S*	R	R	R	R	R
	*rrs* (A907C)	1,472,752	X	X	X	X	/	X	X
	*rrs* (A1401G)	1,473,246	/	/	X	X	X	X	X
	*rpsL* (Lys43Arg)	781,687	X	X	/	/	X	/	/
OFX	Phenotype		R	R	R	R	S*	R	R
MOX	Phenotype		R	R	R	S*	S*	S*	R
GAT	Phenotype		R	R	S*	S*	S*	S*	R
LEV	Phenotype		R	R	R	R	S*	S*	R
FQ gr.	*gyrA* (Ser91Pro)	7,572	X	X	X	X	/	X	X
	*gyrA*(Asp94Gly)	7,582	/	/	/	/	X	/	/
AK	Phenotype		R	R	S	R#	S	S	S
	*rrs*(A1401G)	1,473,246	/	/	X	X	X	X	X
K	Phenotype		R	R	S	R#	S	S*	S*
	*rrs* (A1401G)	1,473,246	/	/	X	X	X	/	/
	*eis* (G-14A promoter)	2,715,346	X	X	X	X	X	/	/
CM	Phenotype		NA	NA	NA	NA	NA	NA	NA
	*rrs* (A1401G)	1,473,246	/	/	X	X	X	X	X
ETO	Phenotype		S	S	S	S	R	S	S
	*inhA* (Ser94Ala)	1674,481	X	X	X	X	/	X	X
PAS	Phenotype		S*	S*	S	S*	S*	S	S
	*folC* (Ser150Gly)	2,747,151	X	X	X	/	X	X	X
	*folC* (Ile43Thr)	2,747,471	X	X	X	X	/	X	X
	*folC* (Glu40Gly)	2,747,480	/	/	X	X	X	X	X

"/"and "X" indicate that a particular SNP was detected or was not detected, respectively, R: resistant, S: susceptible.

*; refers to discordance between susceptible phenotype and resistance genes.

^#^: refers to discordance between resistant phenotype and susceptible (undetected) resistance genes.

Notably, isolates from patient II changed from being susceptible to amikacin (AK) and kanamycin (K) to the resistant phenotype without the detection of known mutations associated with resistance to these drugs.

### Detection of novel mutations putatively associated with drug resistant TB

The comparison between the isolates WBB259 (DS16220) and WBB260 (DS16780) in Patient I showed missense SNPs at genomic position 3,573,234 G>A (CGC>TGC of the minus strand, Arg146Cys) in *Rv3200c* (non-essential gene, possible transmembrane cation transporter [37]) and a reverse missense SNP at genomic position 4,139,678 G>T (AGC>AGA of the minus strand, Ser26Arg) in *glpK* (non-essential gene predicted as probable glycerol kinase in the regulation of glycerol metabolism [37]). The drug-resistance profiles of these isolates were not changed.

**Fig 3:**
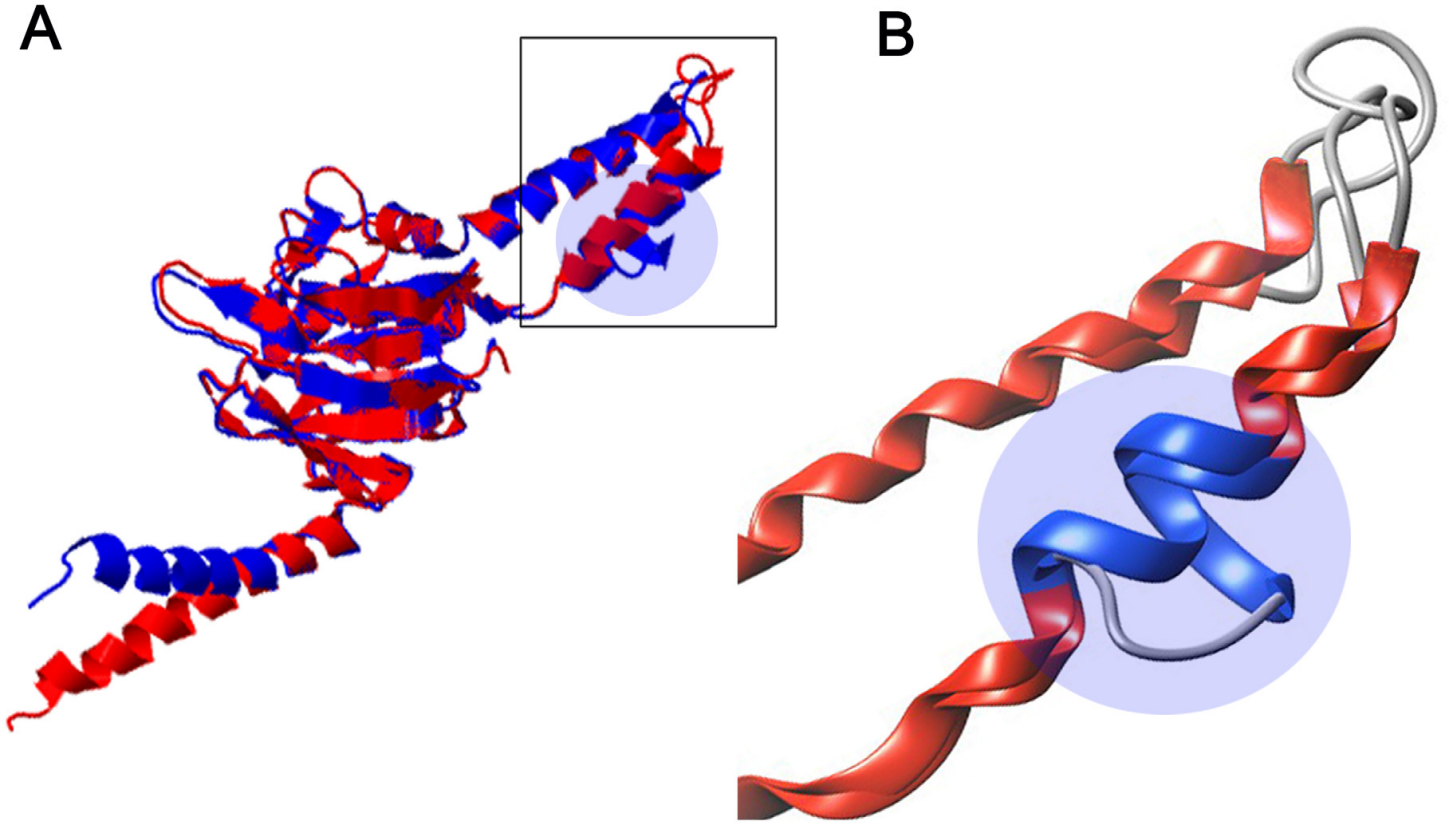
Predicted protein structure *of Rv2477c* affected by the SNP at genomic position 2,783,640 A>C (Trp135Gly). **(A)** Superimposed structures of the 133-amino-acid sequence flanking the SNP. Blue indicates wild-type and red indicates mutant protein. **(B)** Magnified view of the specific protein region affected by SNP of *Rv2477c*. In particular, note the coiled structure (grey rod) in the wild-type (light blue circles) that becomes a helix due to the SNP in the mutant.

Comparison between isolates WBB270 (DS19048) with WBB273 (DS21277) in Patient II showed a silent mutation at genomic position 711,556 C>A (CCC>CCA, Pro7Pro) in *galTa* (non-essential gene, probable galactose-1-phosphate uridylyl transferase [38]) and a missense SNP at genomic position 2,783,640 A>C (TGG>GGG of the minus strand, Trp135Gly) in *Rv2477c* (essential gene, conserved 35 kDa alanine-rich protein predicted to be involved in cell-wall processes and ATP-binding cassette transport systems [39]). The resistance status for kanamycin and amikacin differed between these isolates but no known mutations (*rrs*, *eis* or their promoters from the mutation catalog of tools) associated with resistance against these drugs were detected. The protein structure analysis of *Rv2477c* at genomic position 2,783,640 SNP revealed that glycine had been substituted for tryptophan at amino acid position 135 (W135G), changing the secondary structure from a coiled type into a helix type (Fig 3).

For the comparisons WBB280 (DS29147) vs WBB284 (DS31231) (persistent infection in Patient III), the resistance status for fluoroquinolones (moxifloxacin and gatifloxacin) changed. Missense SNPs were found at genomic position 3,192,484 G>C (TTC>TTG of the minus strand, Phe225Lue) of *pyrH* (essential gene, probable uridylate kinase involved in intermediary metabolism and respiration [40]) and at genomic position 3,339,604 G>A (GGG>AGG, Gly163Arg) of *Rv2983* (non-essential gene, conserved hypothetical alanine-rich protein predicted to be involved in intermediary metabolism and respiration [41]). The *gyrA* mutations were detected by WGS analysis and Sanger sequencing in both WBB280 (susceptible) and WBB284 (resistant) (Tables 1 and 3 and S1 Table).

## Discussion

We analyzed serial isolates of *Mtb* from MDR and XDR-TB patients in Thailand for detection of drug resistance through WGS analysis. We confirm that WGS analysis provides rapid and comprehensive results for molecular epidemiology and detection of drug-resistant TB. A potential marker for kanamycin and amikacin resistance in *Rv2477c* was also identified. We also used the WGS analysis to distinguish between re-infection and persistent infection of MDR and XDR-TB. In the case of reinfection, isolates will differ at many more SNPs than in the case of persistent infection.

Several aspects of the work merit further discussion. First, we analyzed and compared the molecular typing techniques using both classical techniques and *in silico* analysis tools. In a single WGS analysis, molecular typing markers including spoligotyping, LSP and SNP-based typing can be evaluated simultaneously. The robust SNP-barcode typing [35] showed that the strains WBB259 (DS16220) and WBB260 (DS16780) from Patient I belong to lineage 2.1 (non-Beijing in the East Asian (EA) lineage that normally lacks the RD105 deletion), yet had the RD105 deletion. These isolates are rare strains and were defined as SIT523 or the Manu-ancestor lineage. Previously, the clustered isolates based on MIRU-VNTR genotypes were shown to be differentiated based on at least 100 SNPs. WGS analysis provided far higher resolution to differentiate epidemiologically unlinked isolates [42]. In our study, we also noted that the strains with identical 24 MIRU-VNTR genotypes (Patients I and II) could be distinguished using the SNPs identified from WGS analysis.

Previously, admixed infection of two different Beijing isolates from an XDR-TB patient was detected using WGS analysis [12]. Furthermore, WGS analysis of *Mtb* isolates from well-defined outbreaks showed that <11 SNPs occurred per transmission event in the secondary cases [10, 15, 43]. Recently, a cohort study of reinfection and persistent infection isolates of *Mtb* showed that ≤10 SNP and >100 SNPs within approximately 2 years can be used to differentiate between cases of persistent infection and reinfection, respectively [15]. However, the potential applications of WGS to distinguish between persistent infection and reinfection in MDR and XDR-TB patients have never been investigated. We have demonstrated that reinfection and persistent infection in MDR-TB can be distinguished based on the number of SNPs, i.e. 2 SNPs within 8 months for persistent infection vs >60 SNPs within 2 years for re-infection (Patient III). Following from previous studies [15], therefore, a low number of SNPs e.g. <10 SNP can indicate persistent infection in M/XDR-TB, while >60 SNP differences are likely to indicate reinfection. Notably, the SNPs identified in our study and previous studies were high-confidence SNPs based on comparable filtration criteria (≥0.75 allele frequencies, ≥15.6% cutoff of average sequencing depth). In Patient II, there were 2 SNPs between serial isolates within 2 months. This is relatively high compared to findings of previous studies and might be due to high selective pressures on the MDR and XDR isolates studies and the high mutation rate of the Beijing lineage [16] under selective pressure of antituberculous drugs used. We also analyzed drug resistance genes in these serial *Mtb* isolates. We used several bioinformatics tools to increase sensitivity for detecting markers for drug resistance. The performance of the tools was different. However, our sample population is too small to be used for a comprehensive evaluation of these tools. Based on *in silico* tools, WGS analysis can detect known genetic markers associated with resistance to streptomycin, fluoroquinolones and *p*-aminosalicylic acid in isolates with a susceptible phenotype to these drugs. WGS, therefore, seems more sensitive for detection of drug-resistant TB compared to phenotypic tests. This possibility is supported by the influence of the borderline-susceptible or low-level resistance close to the sensitive/resistant break point leading to discordance between mutation and DST [44]. Alternatively, these known genetic markers associated with drug resistance in susceptible phenotypes are not high-confidence SNPs validated using large global cohorts [1]. Furthermore, the possibility of false positive SNPs detected in our analysis should be acknowledged. Only drug resistance-associated SNPs in *rrs* (for streptomycin) and *gyrA* (for fluoroquinolones), detected in those DST-discordant strains, are high-confidence SNPs based on published information [27, 29, 30, 45]. Furthermore, we found that the known SNPs in *gyrA* did not correlate with resistance to all fluoroquinolones but primarily to ofloxacin. This finding suggests that different mutations in *gyrA* are associated with resistance against different drugs in the fluoroquinolone group, as indicated by previous study [46]. Notably, the phenotypic DST in our study was mainly done using the agar-based test that can be confounded by protocol variation e.g. in preparation of inoculums, leading to discordant result [44]. Nonetheless, the phenotypic DST results in our study were repeated to ensure the reliable phenotypic results.

Although catalogs of genetic markers associated with drug-resistant TB have been developed [27, 29, 30, 45], several genetic markers, especially those associated with second-line drug resistance, remain unknown. In our study, isolates from Patient II and Patient III exhibited changes in drug susceptibility that can be analyzed for drug resistance genes. Interestingly, isolates from Patient II changed from the kanamycin and amikacin-susceptible to the resistant phenotype without known genetic markers being detected. These isolates provide a tool for identifying possible novel kanamycin and amikacin resistance genes. One known resistance mechanism to kanamycin and amikacin is a mutation of *rrs* (encodes 16S rRNA) which confers 30–90% resistance to kanamycin [47]. Another known mutation of kanamycin is in *eis* (aminoglycoside acetyltransferase-encoding gene) [48]. We found that a high-confidence SNP at genomic position 2,783,640 A>C of *Rv2477c* might be a novel candidate associated with kanamycin and amikacin resistance. Based on the full length of *Rv2477c* (558 amino acids), the non-synonymous SNP at position 2,783,640 A>C alters the amino acid at position 135 from tryptophan into glycine. As glycine has a smaller size and higher hydrophobicity than does tryptophan, such an alteration might affect the structure and function of *Rv2477c*. Indeed, this SNP changes the secondary structure of this protein from the coiled type into the helix type and changed the adenosine diphosphate (ADP) binding site of *Rv2477c* in the mutant (data not shown). The function of *Rv2477c* is still unclear but it may be an essential gene involved in cell wall processes and efflux pumping-associated ABC transport systems [39]. Previously, the proteomic comparison of kanamycin-susceptible and resistant strains revealed that the quantities of several proteins were increased in the resistant strains and that *Rv2744c* is one of those [49]. Furthermore, microarray study revealed over-expression of *Rv2477c* in multi-drug resistant *Mtb* isolates [50]. Therefore, a functional study investigating the mutation of *Rv2477c* and drug resistance to kanamycin and amikacin is warranted.

In Patient III (WBB280 (DS29147) vs WBB284 (DS31231)), the drug-resistance profiles of fluoroquinolones (moxifloxacin, gatifloxacinand and levofloxacin) changed from susceptible to resistant. Although missense SNPs of *pyrH* and *Rv2983* were found in the latter isolate, the known *gyrA* mutation was also detected by WGS analysis and Sanger sequencing in both isolates. Thus, the SNP of *pyrH* and *Rv2983* might be a spontaneous mutation, or a candidate mutation associated with fluoroquinolone resistance, i.e. mutation of *gyrA* Asp94Gly is not seen in all resistant phenotypes. In Patient I, although 2 missense SNPs were detected between 2 serial isolates, no change in drug susceptibility profiles were detected. These mutations might reflect only background spontaneous mutation or other mutations not involved with drug resistance.

We found conversion of the moxifloxacin-resistant phenotype to the susceptible phenotype in Patient II. A possible explanation is that a mutation conferring resistance to one drug can influence responses to other drugs, causing such a change. For example, development of resistance to colistin in *Acinetobacter baumannii* seemed to have led to increased susceptibility to other antibiotics [51]. Alternatively, the mixed populations of susceptible and resistant bacilli to moxifloxacin might affect such a phenotypic conversion [52].

In summary, we demonstrated that WGS can be used to distinguish between reinfection and persistent infection TB events in MDR- and XDR-TB strains using cutoff numbers of SNPs. A novel candidate mutation associated with aminoglycoside (kanamycin and amikacin) resistance in *Rv2477c* was suggested. We also demonstrated that WGS analysis using various *in silico* analysis tools had the following advantages in TB study; (i) early and rapid detection of drug-resistant TB, (ii) prediction of drug resistance not revealed by phenotypic DST (but note that low-confidence SNPs from the mutation catalog might not actually be associated with a drug-resistant phenotype), (iii) provision of information for molecular epidemiology of TB such as spoligotyping, LSPs and SNP-base typing. Other useful information regarding drug-resistance genes and molecular epidemiology of TB was also provided.

## Supporting Information

S1 Table Drug resistance associated mutations detected using bioinformatics tools. (XLSX)
